# Celiac Disease and Non-celiac Wheat Sensitivity: State of Art of Non-dietary Therapies

**DOI:** 10.3389/fnut.2020.00152

**Published:** 2020-09-08

**Authors:** Gloria Serena, Paolo D'Avino, Alessio Fasano

**Affiliations:** ^1^Division of Pediatric Gastroenterology and Nutrition, Center for Celiac Research, Mucosal Immunology and Biology Research Center, Massachusetts General Hospital, Boston, MA, United States; ^2^Vita-Salute San Raffaele University, Milan, Italy; ^3^European Biomedical Research Institute of Salerno (EBRIS), Salerno, Italy

**Keywords:** celiac disease, gluten sensitivity, gluten, therapy, treatment

## Abstract

Gluten related disorders (GRD), which include celiac disease, non-celiac wheat sensitivity and wheat allergy are heterogeneous conditions triggered by ingestion of gluten-containing grains. Together, their prevalence is estimated to be ~5% in the general population, however, in the last years the number of diagnoses has been rapidly increasing. To this day, the gold standard treatment for these disorders is the complete removal of gluten-containing grains from the diet. Although this therapy results effective in the majority of patients, up to 30% of individuals affected by GRD continue to present persistent symptoms. In addition, gluten-free diet has been shown to have poor nutritional quality and to cause a socio-economic burden in patients' quality of life. In order to respond to these issues, the scientific community has been focusing on finding additional and adjuvant non-dietary therapies. In this review, we focus on two main gluten related disorders, celiac disease and non-celiac wheat sensitivity. We delineate the actual knowledge about potential treatments and their relative efficacy in pre-clinical and clinical trials.

## Introduction

The term “gluten-related disorders” (GRD) describes an array of diseases, including celiac disease (CD), wheat allergy (WA), and non-celiac wheat sensitivity (NCWS), for which gluten is considered the main external trigger. Gluten is a complex molecule found in different grains such as wheat, rye, and barley (1). Its protein fraction includes gliadin monomers and glutenins polymers, both resistant to complete degradation in the gastrointestinal tract due to their high content of prolines and glutamines (2, 3). Human exposure to gluten started around 10,000 years ago with the advent of agriculture and the first description of CD has been attributed to the ancient Greek physician Aretaeus (4). The overall incidence of gluten related disorders has been quickly increasing in the recent years (5), therefore making them an important topic of new scientific discussions (6) and pushing the scientific community to deeply explore their pathogenesis.

Given the recognized role of gluten as external trigger for CD, WA, and NCWS, a life-long removal of this protein from the diet is the gold standard treatment for people affected by these conditions (1). The need of alternative and/or adjuvant therapies, however, has been highlighted by recent studies underlying the economic burden and the poor quality of life associated with this treatment (7, 8) as well as the overall decreased nutritional quality of gluten free products as compared to their gluten containing counterparts (9). In this review we will focus on two gluten-related disorders, CD and NCWS, for which additional therapies other than a gluten-free diet have been considered. We will examine the pathogenic cascade of each condition (summarized in Table 1) and describe ongoing and past clinical trials using alternative therapies to target different steps of this process (Summarized in Table 2).

**Table 1:** Main features of celiac disease and non-celiac wheat sensitivity.

	**CD**	**NCWS**
Definition	Autoimmune disease characterized by aberrant response to gluten in genetically susceptible individuals	Reaction to gluten that is not mediated by an allergic or auto-immune response
Prevalence	~1%	Between 3 and 6%
Genetic background	HLA DQ2/DQ8	Only 50% of patients carry HLA-DQ2/ or HLA-DQ8 haplotype
Pathogenesis	Innate and adaptive immune response (IL15, IL8, IELs, Th1/Th17, antibodies production), increased intestinal permeability	Altered expression of TLR1,2,4, reduction in Tregs clones expansion
Diagnosis	• Serological evaluation for Ig anti-TTG • Duodenal biopsies is gold standard	Six weeks of GFD with evaluation of symptoms and 2 weeks of placebo/gluten challenge

**Table 2:** Past and ongoing clinical trials on CD and NCWS patients as reported by clinicaltrials.gov. Status of the trials is shown as completed (C), active (A), recruiting (R), or unknown (U).

**Compound**	**Celiac disease**	**Non-celiac-wheat sensitivity**
Endopeptidases	NCT01560169 (ALV003) (C)	NCT02060864 (AN-PEP) (C)
	NCT00859391 (ALV003) (C)	
	NCT00669825 (ALV003) (C)	
	NCT01255696 (ALV003) (C)	
	NCT01917630 (ALV003) U	
	NCT00626184 (ALV003) (C)	
	NCT00959114 (ALV003) (C)	
	NCT00962182 (STAN1) (C)	
	NCT00810654 (AN-PEP) (C)	
Gluten sequestring polymer	NCT01990885 (BL-7010) (C)	
Tight junction modulators	NCT00386490 (LARAZOTIDE) (C)	
	NCT03569007 (LARAZOTIDE) (R)	
	NCT00889473 (LARAZOTIDE) (C)	
	NCT00362856 (LARAZOTIDE) (C)	
	NCT00492960 (LARAZOTIDE) (C)	
	NCT00386165 (LARAZOTIDE) (C)	
	NCT00620451 (LARAZOTIDE) (C)	
	NCT01396213 (LARAZOTIDE) (C)	
TG2-inhibitors	2017-002241-30 Clinical Trials Register European Union (ZED 1227) (C)	
Immune therapies	NCT01893775 (Mik-β-1) (C)	
	NCT02637141 (AMG714) (C)	
	NCT02633020 (AMG 714) (C)	
	NCT00540657 (CCX282-B) (C)	
Necator americanus	NCT01661933 (C)	
Nanoparticles	NCT03486990 (CNP-101) (C)	
Gluten vaccine	NCT03644069 (NexVax2) (A)	
Probiotics	NCT03176095 (*L.plantarum,L. paracesei*) (C)	NCT02810301 (ES1_*B.longum*) (U)
	NCT03775499 (*B. longum*) (A)	NCT03775499 (*B.longum*) (A)
	NCT04160767 (VivoMixx probiotic mix) (R)	
	NCT01257620 (*B.infantis*) (C)	
	NCT01699191 (*L.plantuarum, L. casei, B. breve, B. animalis*) (U)	
	NCT03562221 (*Lactobacilli*) (R)	
	NCT03857360 (Pentabiocel probiotic mix) (R)	
	NCT04014660 (*L.plantarum, L.paracesei*) (R)	
	NCT03271138 (*B.infantis*) (C)	
	NCT02244047 (*B.breve*) (C)	

### Celiac Disease (CD)

**Figure 1:**
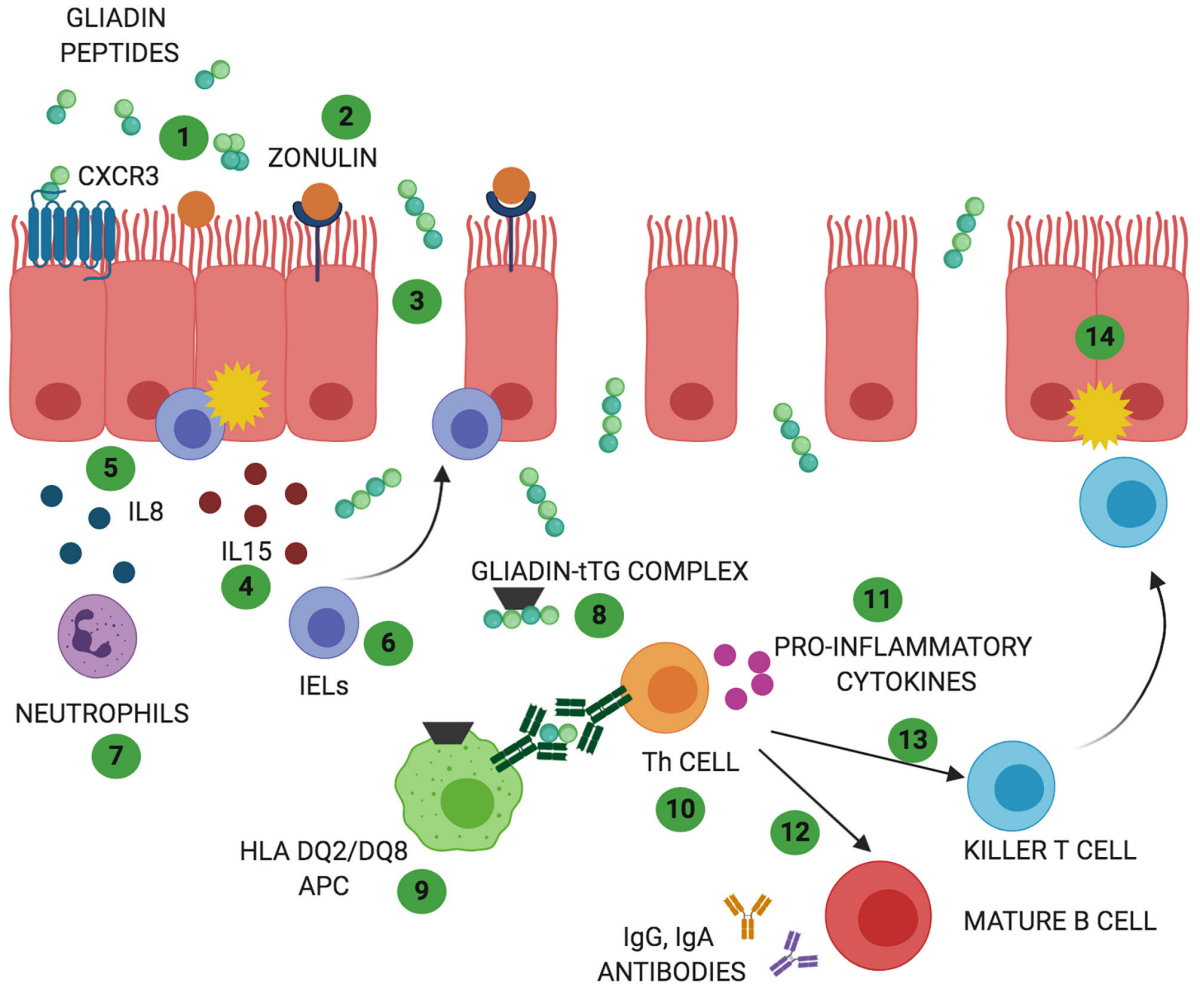
Celiac disease (CD) and non-celiac wheat sensitivity (NCWS) pathogenesis: In both gluten-related disorders partially digested gliadin peptides interact with the epithelium and trigger an immune response. In CD these peptides interact with CXCR3 receptor expressed on the epithelium (1) triggering release of zonulin and leading to an increase in intestinal permeability (2). In NCWS the involvement of CXCR3 or zonulin has not been reported but a decrease in intestinal permeability has been described. Due to the functional loss in gut barrier function, in CD, gliadin peptides translocate to the lamina propria (3). Here, they trigger an innate immune response characterized by release of IL15 (4) and IL8 (5) with consequent activation of IELs (6) and recruitment of neutrophils (7). Activation of innate immune response with increased IELs has been reported also in NCWS patients. However, the exact players involved in this gluten related disorder are still unknown. In CD, the innate immune-mediated response is followed by release of tissue transglutaminases 2 (TG2) that deamidate gliadin peptides (8) allowing their recognition by HLA DQ2/DQ8 antigen presenting cells (APC) (9). In NCWS the role of TG2 has not been described and the HLA haplotype DQ2/DQ8 is present only in 50% of the patients. In CD, antigen presentation by APC results in T helper cells activation (Th1 and Th17) (10). Th cells produce pro-inflammatory cytokines (11) that trigger maturation of IgG and IgA producing B cells (12) and activation of killer T cells (13) ultimately leading to intestinal damage (14). While activation of a chronic adaptive and humoral immunity represents a main feature of CD pathogenesis, they do not appear to characterize NCWS patients.

CD is a complex autoimmune disease triggered by ingestion of gluten. It is characterized by a specific genetic predisposition (HLA DQ2 and/or DQ8) and a continuous exposure to the external trigger (gluten) that leads to increased intestinal permeability and a chronic immune response (9). CD affects around 1% of population worldwide (9). Its classic clinical presentation includes gastrointestinal symptoms such as diarrhea, malabsorptive manifestations, constipation, and abdominal pain. In addition, extra intestinal symptoms such as anemia, neurological symptom, dermatitis, and arthritis may be present as well, in particular among adult patients (10). CD pathogenesis has been studied for long time and the events by which gliadin triggers the onset of the disease are hypothesized as following (Figure 1): due to its unique aminoacidic composition, gliadin can only be partially digested in the gastrointestinal tract and the resulting peptides trigger an innate immune response by physically interacting with the small intestinal mucosa. As a result, CD8^+^ intraepithelial lymphocytes (IELs) proliferate, several cytokines such as IL8 and IL15 are secreted by enterocytes and dendritic cells, neutrophils are recruited in the lamina propria and enterocytes apoptosis is induced by activation of NKG2D^+^ cells (11–16). Activation of CXCR3 receptor on the epithelium triggers secretion of zonulin, a potent tight junction modulator, leading to increased intestinal permeability, and exaggerated antigen trafficking through the intestinal barrier (17, 18). Once translocated in the lamina propria, gliadin peptides are deamidated by transglutaminase2 (TG2) (19) and acquire a strong affinity for HLA DQ2^+^ and DQ8^+^ antigen presenting cells (APC), that will later activate a Th1/Th17 driven adaptive immune response characterized by production of IFNγ, TNFα, IL18, IL21, and IL17 (16). These pro-inflammatory cytokines further increase the intestinal permeability and damage in the intestinal mucosa. As a result of cooperation with gluten-reactive T cells, B cells differentiate into plasma cells and secrete anti-tTG2 and anti-gliadin antibodies (20). Finally, continuous ingestion of gluten, paired with inefficient suppressive regulation by Treg cells, leads to chronicity of the immune response (21, 22).

CD diagnosis includes small intestinal biopsy and serological tests. The endoscopy should show histological changes such as increased number of intraepithelial lymphocytes, elongated crypts, and villous atrophy. Serological exams should detect anti-tTG IgA and IgG as well as anti-endomysial IgA antibodies (10).

For long time CD has been considered a pediatric condition; however the recent increase in diagnoses among adult patients and longitudinal prospective studies suggest that tolerance to gluten can be lost at any time in life (23). These data, together with the overall increase in CD incidence that has been characterizing the last decades, indicate that additional factors, other than genetic predisposition and external trigger, may contribute to the onset of the disease (23). Environmental changes and alterations in the microbiome composition are considered strong candidate factors as they appear to have a potential role in triggering CD onset.

To this day, complete elimination of gluten from the diet (GFD) is the gold standard treatment for CD (24), in that it allows clinical and histological recovery within 1 year and prevents long-term complications. The burden of a life-long gluten free diet in CD patients, however, has been described by several studies (7, 8). Adherence to gluten free diet has been reported to be as low as 36–45% (25–27) with limited availability, high cost of gluten free products and social isolation being raised as main causative factors of this poor adherence (28–31). A lack of proper treatment in CD subjects can lead to important complications such as decreased bone density, increased risk of malignancies and higher mortality rate (32, 33).

The above listed complications as well as many others have been described in a sub-population of CD patients affected by non-responsive celiac disease (NRCD) that present persistent symptoms despite 6–12 months of gluten-free diet (34). While NRCD can be related to alternative primary diagnosis and associated conditions, a small percentage of patients are diagnosed with refractory celiac disease (RCD) (35). RCD is defined as a persistent mal-absorptive condition triggered by ingestion of gluten with symptoms that persist despite a strict gluten free diet (36). RCD can affect approximately between 0.3 and 4% of CD patients and it can be sub-classified in RCD1 or RCD2 depending on the abnormal expansion of a specific subset of IELs and severity of symptoms (36, 37). RCD1 patients present mild symptoms and show good response to treatment with 80–100% of 5 year-survival; on the contrary symptoms in RCD2 patients are severe and only 50% of these individuals receive a 5-year prognosis with mortality occurring as a result of malnutrition and development of associated complications (36).

Overall, the increasing incidence in CD and RCD diagnosis over the last years combined with the burden of following a gluten free diet, highlighted the need of alternative and/or adjuvant therapies for CD.

### Non-celiac Wheat Sensitivity (NCWS)

In addition to CD, non-celiac wheat sensitivity (NCWS) is another gluten-related disorder whose incidence is increasing over the years (38). It is defined as a non-allergic, non-autoimmune condition characterized by intestinal and/or extra-intestinal symptoms resulting from gluten-containing grains, that resolve once these grains are removed from the diet (2, 39, 40).

NCWS affects between 3 and 6% of the general population with a higher incidence in females than in males (41). However, exact epidemiological and prevalence data about this condition are still missing due mainly to the fact that the majority of patients start to follow a gluten-free diet without having received a diagnosis from a physician. Conversely to CD, NCWS has not been associated to a strong genetic predisposition. Indeed, only 50% of NCWS patients express the HLA-DQ2 and/or HLA-DQ8 haplotype (2), therefore suggesting that these genes are not necessary or sufficient to develop the condition. Furthermore, while for CD association with non-HLA genes has also been reported (42, 43), the same has not been described for NCWS.

NCWS clinical presentation includes both intestinal (stomach pain, diarrhea, bloating) and extra-intestinal symptoms (anxiety, depression, fatigue, rash, headache, “foggy brain,” joint and muscle pain, and anemia) (2, 39). All symptoms usually occur soon after wheat-ingestion and improve or disappear within hours or few days after stopping the assumption of wheat.

NCWS pathogenesis is not yet completely understood and it differs from CD pathogenesis in many ways (44, 45). It is known to start when the gut epithelium enters in contact with gluten storage proteins eliciting an immune response (45). Mucosa of NCWS patients presents increased CD3^+^ alpha/beta IELs compared to healthy controls but their number is significantly lower than the one recorded in active CD (39, 46). Furthermore, unlikely CD, NCWS patients do not show apoptotic enterocytes or villous atrophy (47). Another main difference between NCWS and CD pathogenesis is the lack of adaptive Th1/Th17 driven immune response (38) while several studies have suggested an important role of the innate immunity in the pathogenesis of this condition (38, 39, 47). In addition, impaired regulation of the immune response characterized by reduction in Treg clones expansion and their cytokines production (TGFβ1 and IL10) has been reported to contribute to NCWS pathogenesis as well (38). Sapone et al. have shown an increased expression of TLR, especially TLR1, 2, 4 in NCWS as well as a decreased intestinal permeability (assessed by lactulose-mannitol test) (38).

To this day there is no reliable biomarker for NCWS and its diagnosis is based on exclusion criteria only (2, 48). In order to be diagnosed with NCWS a patient should be symptomatic and the symptoms should improve within few days after starting a gluten-free diet. In addition, the patient should be excluded for other conditions (CD, WA, type 1 diabetes mellitus, inflammatory bowel disease, *Helicobacter pylori* infection) and test negative for skin prick test for wheat or autoantibody serology (EMA-IgA and tTG-IgA). Finally, intestinal endoscopy should show normal mucosa (Marsh 0–1). To confirm NCWS diagnosis physicians should require patients to start a 6 weeks period of GFD during which the symptoms are evaluated, followed by 2 weeks of placebo/gluten challenge (44, 49, 50).

As seen for other gluten related disorders, NCWS incidence is rapidly increasing. However, given the heterogeneity of symptoms and the challenges encountered by scientists in understanding its pathogenesis, reliable biomarkers for NCWS are still missing. In recent years the role of gluten as main trigger for NCWS has become a matter of debate as it has been described that, beside gluten, amylase trypsin inhibitors (ATI), and fermentable oligo- and disaccharides, monosaccharides, and polyols (FODMAPs) may also been involved in inducing abdominal symptoms typical of NCWS. In light of this, the scientific community has started to propose new adjuvant and/or alternative therapies to GFD. The use of different approaches in treating NCWS patients would not only benefit their quality of life, but would also give important insights on the pathogenesis of this disease.

## Alternative/Adjuvant Therapies to Gluten Free Diet

Since 2005 several clinical trials have started focusing on different targets with the final goal of preventing the chronic immune response triggered by gliadin peptides in individuals affected by CD or NCWS (Table 2). In addition preclinical studies focused on new technologies are also being developed in the attempt to target other steps of the pathogenic cascade of these gluten related disorders.

### Endopeptidases

The lack of complete digestion by gastro-intestinal enzymes is one of the main features of gliadin. The resulting partially digested peptides have been shown to have cytotoxic, immunomodulatory and barrier modulating activities (51) therefore contributing to CD onset. Based on this unique gliadin characteristic, the use of different endopeptidases has been tested in several clinical trials to further digest gliadin peptides and neutralize their toxic activity.

Latinoglutenase (previously called ALV003) is an orally administered combination of two different proteases that decrease the immunogenic potential of gluten proteins by degrading them (ALV001, an EP-B2 cysteine endopeptidase) and by catalyzing the post-proline hydrolysis of proteins and peptides (ALV002, a prolyl endopeptidase) (52, 53). A phase zero clinical trial found that pre-treatment with ALV003 (16 g daily for 3 days) inhibits IFNγ production by peripheral T cells from CD patients in response to 33mer gliadin derived peptides (54). Two separate phase 1 clinical trials using single, escalating doses of ALV003 (100, 300, 900, and 1,800 mg) confirmed its stability in the stomach, its capability of degrading dietary gluten and confirmed the lack of adverse events or allergic reactions (55). Finally, a phase 2 trial, in which CD patients were challenged for 6 weeks with gluten, demonstrated the efficacy of ALV003 (900 mg) of attenuating mucosal injury, but showed no improvement in symptoms (56). Another more recent phase 2 trial did not find any differences between placebo and Latinoglutenase treatments (100, 300, 450, 600, and 900 mg) in term of IELs number, villous height:crypt ratio or serological markers (57).

A 2 weeks long randomized, double-blinded, placebo-controlled study performed on 16 CD patients challenged with 7 gr of gluten/day, showed that AN-PEP, a prolyl peptidase derived from *Aspergillus niger*, was well-tolerated and that it improved patients' quality of life. However, the same study also found no differences in duodenal mucosal changes between the groups (58, 59). Given the short time period challenge and the scarce number of patients, more trials are needed to understand the efficacy of this enzyme.

The efficacy of AN-PEP in degrading gluten has also been tested in NCWS patients (60). In this double-blinded, placebo controlled study both low (80,000 PPI) and high doses (160,000 PPI) of AN-PEP were shown to be effective in reducing gluten concentrations in the stomach and duodenum. While this trial was not designed to evaluate the safety of the endopeptidase, no adverse events were reported.

A third endopeptidase that has been explored is STAN-1, a cocktail of microbial enzymes (0.7 mg/ ml of dipeptidyl peptidase IV and 0.35 mg/ml of asperegillopepsin) specifically designed to degrade gluten in the gastrointestinal tract. A randomized, double-blind placebo controlled trial performed on 35 patients with persistent elevated tTG-IgA and challenged for 12 weeks with 1 gr of gluten daily, however, did not show any differences in tTG-IgA (61).

Finally Kuma030 is an engineered serine-endoprotease derived from acid collagenase and produced by *Alicyclobacillus sendaiensis* with an increased proteolytic activity and a higher specificity for gliadin peptides (62). A pre-clinical study has shown promising results with Kuma030 reducing IFNγ production and T cells proliferation in a dose dependent manner (62).

Overall endopeptidases appear to be effective in digesting small quantities of gluten, but not larger amounts, therefore placing them as possible adjuvant therapies, but not alternative therapies to the gluten free diet (54, 63).

### Gluten Sequestering Polymers

Gluten sequestering polymers technology relies on the capability of these compounds of sequestering intraluminal gliadin in order to prevent its breakdown into immunogenic peptides (64). BL-7010 is a synthetic, non-absorbable copolymer that, once tested in preclinical trial in NOD-DQ8 mice (3,000 mg/kg administered by oral gavage), has shown to prevent reduction of villous to crypt ratio, alter barrier function and intraepithelial (64). While a phase I/II trial looking at the safety of the polymer has been completed at Tampere University, in Finland, in collaboration with BioLineRx Ltd., data have not been published yet.

### Larazotide Acetate

Altered intestinal permeability is one of the main features characterizing CD and NCWS pathogenesis (65). Zonulin-dependent disruption of tight junctions architecture, in fact, enhances antigen trafficking and translocation of gliadin peptides into the intestinal lamina propria (20). Based on this knowledge, the concept of using tight junction modulators able to limit this effect is an attractive one. Larazotide acetate (also called AT1001) is a synthetic octapeptide with a high homology sequence with larazotide and its *Vibrio cholerae* toxin zonula occludens analog (66). Preclinical studies in intestinal cell lines (MCDK, CaCo2, and IEC6) as well as in HLA-HCD4/DQ8 double transgenic mice have shown the efficacy of AT1001 in restoring overall intestinal barrier function by reducing gliadin induced intestinal permeability, inflammatory cytokines release and gliadin translocation (67, 68).

A phase I, double-blind randomized placebo study performed on 20 CD patients in an inpatient setting demonstrated that Larazotide (12 mg) is safe and well-tolerated. Furthermore, it also detected differences in intestinal permeability between the placebo and treatment groups (66). Those promising results concerning changes in intestinal permeability were not confirmed by another placebo-controlled study involving 86 patients treated in an outpatient setting with different doses (0.5, 1, and 2 mg administered three times a day) of the tight junctions modulator (63). Interestingly, the same study found a reduction of symptoms severity after low doses of Larazotide. Similar results were reported in a subsequent clinical trial in which 184 patients were treated for 6 weeks with different doses of Larazotide (1, 4, and 8 mg three times a day) or placebo (69). Finally, a clinical trial performed on 342 non-responsive CD patients confirmed the effectiveness of low doses of Larazotide (0.5 mg) in significantly reducing symptoms compared to the placebo group. Given these promising results, a phase 3 clinical trial has been approved by the Food and Drug Administration (FDA) and it is now ongoing for the treatment of non-responsive CD (70).

Overall the data collected by these studies suggest that Larazotide acetate may be effective as adjuvant therapy used to tolerate minimal amounts of gluten, rather than as alternatives to a GFD. Furthermore, given its mechanism of action it could be tested also in other conditions in which altered intestinal permeability plays a role such as NCWS.

### Transglutaminases 2 Inhibitors

Transglutaminases 2 (TG2) play a major role in CD pathogenesis. They are responsible for gliadin peptides deamidation and trans-deamidation, therefore leading to the presentation of these peptides by HLA DQ2 and/or DQ8 APC to T cells. To this day, two major types of TG2 inhibitors have been proposed: competitive and irreversible. The first ones block the substrate access to TG2 active site by adopting competitive mechanisms, while the second ones cause covalent modification of TG2 by bind irreversibly to them (70). Both *in vitro* and *ex vivo* studies performed on CaCo2 cells and duodenal biopsies have shown the efficacy of two TG2 inhibitors (R281 and R283) in reducing gliadin induced toxic effects (71, 72). Despite the promising results, TG2 inhibitors could lead to severe side effects in that TGs are ubiquitously expressed in human tissues and their shared high-homology sequences may become undesired targets for these compounds (63).

New generation TG2 inhibitors (ZED1098, ZED1219, and ZED 1227) have been developed to have high affinity for intestinal TG2 and to overcome the side effects that R281 and R283 may have. Preclinical studies on mice have confirmed the capability of these new inhibitors of inhibiting intestinal TG2, while a phase 2 clinical trial is still ongoing (70). Given the fundamental role that TG2 play in regulating intestinal inflammation and healing, the safety and efficacy of these inhibitors need to be further tested.

### HLA DQ2/DQ8 Blockers

The HLA haplotype DQ2 and/or DQ8 represent a genetic predisposition necessary for CD development with T cells presentation of gliadin peptides by HLA DQ2/DQ8 APC being a crucial step of CD pathogenesis. In their study, Kapoerchan et al. have studied the binding properties of previously identified natural ligands for HLA DQ2 and, through substitution analysis, they created peptides with an affinity for HLA DQ2 that were 200 folds higher than gluten derived peptides (73). Some of these peptides, were found to be non-immunogenic and to block gluten induced immune response (73). While conceptually promising, the use of HLA blockers has raised several issues and pre-clinical trials have been yet to be initiated. Due to the partial agonist effect exerted by these peptides, in fact, HLA DQ2/DQ8 blockade may not be complete, therefore resulting in severe immunosuppression (70, 74).

### Immune Therapies

Given the important role that the immune response plays in CD and NCWS pathogenesis, immune therapies have been often considered as possible adjuvant therapies to GFD. Among them, IL15 antagonist is, so far, the most promising one. In CD patients, IL15 secretion by enterocytes and APC leads to increased lymphocytosis and inhibition of IELs apoptosis (75). Furthermore, IL15 has also been shown to suppress Treg cells function in CD patients (76). Based on these evidences, IL15 signaling blockade has been considered a possible therapeutic target in particular for RCD patients, for whom lymphocytosis represents a persistent symptom.

To this day, three main IL15 antagonists have been tested in pre-clinical and clinical trials. Tofacitinib is a pan-JAK inhibitor already approved by the FDA for rheumatoid arthritis. It has been proven to be effective in blocking IL15 and in reversing pathological manifestations in a mouse model for CD (77). Its use in clinical trials for RCD is now being considered. Humanized Mik-β-1 is a monoclonal antibody specific for CD122 (IL2/IL15 receptor) currently tested in a phase 1 clinical trial in patients with RCD. Another monoclonal antibody, AMG 714 is under investigation in a phase 2 clinical trial in RCD type 2/pre lymphoma and in non-responsive CD patients. Although in concomitance with adverse events such as pneumococcal infection, hypertransaminasemia, gait disorder, tuberculosis, and cerebellar syndrome, its intravenous use at 8 mg/kg dose has been shown to be effective in improving symptoms and reducing IELs progression in RCD type 2 patients (78). Similarly, another phase 2a clinical trial tested different doses of AMG 714 in 64 non-responsive CD and found a significant symptoms improvement as well as IELs reduction (79).

CCR9 is a chemokine receptor fundamental for T cells migration into the intestinal compartment (80). As such, the use of CCX282-B, a CCR9 receptor antagonist, has been considered as an alternative therapy for CD patients. A phase 2 clinical trial has been performed in CD patients following a GFD and challenged with gluten, however, the results have not been published yet (74).

### Necator americanus

The immune system is a complex machinery in which homeostasis is maintained through a delicate balance between different types of immune response (81), therefore, Th2 regulated parasitic infections have been suggested to prevent the onset of Th1 driven autoimmune diseases (82). Based on this assumption, the effect of hookworm *Necator americanus* has been investigated on 12 CD patients micro-challenged with gluten for 52 weeks (83). This study showed an improvement in patients' quality of life and a reduction of mean tTG IgA titers, however the villous height-crypt ratio did not decrease. A randomized double-blind placebo controlled study has tested the effect of hookworm *Necator americanus* on CD patients that had been challenged with gluten for 21 weeks (84, 85). The study did not show any difference in histological damage or number of IFNγ producing cells. Furthermore, while infected patients showed a decreased production of IL7a and IFNγ, no hookworm effect was detected in response to anti-gliadin peptide lymphocytes. Results from the same clinical trial continued for extra 12 weeks are still to be published. Although the idea of adopting helminths infections as a treatment for CD and other autoimmune diseases such as IBD and multiple sclerosis has been suggested for long time, major concerns are still making their clinical use challenging. The exact mechanisms through which helminths are able to suppress autoimmune response are still unknown (86) and the effects provoked on patients by a long exposure to helminths infections are unpredictable (87). Thus, before adopting *Necator americanus*, as an alternative therapy to GFD, more pre-clinical studies have to be performed.

### Other Adjuvant Therapies

The use of nanoparticles or cathepsin s inhibitor has been proposed as an adjuvant treatment for CD. CNP-101 (formerly TIMP-GLIA) is a gliadin protein embedded in polymer matrix of poly DL-lactide-coglycolide nanoparticles that, through a non-inflammatory antigen presentation, is suggested to develop an immune tolerance to gluten. A phase 2 double blind randomized proof of concept study has shown the efficacy of CNP-101 in reducing IFNγ response, while no differences in villous height:crypt depth ratio were reported (70).

Cathepsin is a lysosomal cysteine protease important for antigen presentation by MHC molecules (88). A randomized, phase 1 double blind placebo-controlled multiple dose study has focused on a specific cathepsin s inhibitor (R05459072) and investigated its efficacy, safety, tolerability, and pharmazokinetics/dynamics in patients affected by CD that followed a strict GFD. The study has been completed, but results are yet to be published.

An aberrant immune response plays a fundamental role in CD pathogenesis, therefore the use of an immunotherapy to treat the disease has been suggested for long time. NexVax2 is a peptide-based, epitope specific gluten tolerizing agent that recognizes the HLA DQ2.5 epitope TCR complex (89) and that has been proposed as an alternative therapy for CD.

A phase 1 clinical trial monitoring tested NexVax2 safety in 34 patients with CD and highlighted some important side effects such as abdominal pain and vomiting (90). Other two phase 1 trials showed adverse acute gastrointestinal side events that were similar to gluten ingestion (90). A later study showed improvement in duodenal mucosal damage in a limited number of patients (91). A phase 2 clinical trial was due to results showing no differences between the vaccine and the placebo in protecting patients from gluten exposure. While the use of immunotherapies such as NexVax2 is theoretically promising, a better understanding of its safety and efficacy is still needed.

### Probiotics

The gut microbiota is constituted by a complex community of microorganisms residing in the host gastrointestinal tract (92). Despite the microbiota being stable throughout the life of a healthy person and reaching the total colonization of the gut by 3 years-old, some elements can affect its composition. The main factors that can modulate gut microbiota are host diet, hygiene, drugs, and stress. Additionally also infectious, metabolic and inflammatory diseases can also deeply perturb the gut microbiota (93–95). The alteration of commensal microflora composition is known as, dysbiosis.

Intestinal commensal bacteria and their metabolites play a major role in maintaining host overall health. They are able to modulate intestinal barrier function and contribute to the development of protective/tolerogenic immune response (96, 97). The correlation between CD and intestinal dysbiosis has been described by several groups (98–100). Indeed, gut microbiota has been shown to contribute to CD pathogenesis by (1) controlling the digestion of gluten peptides therefore generating toxic and tolerogenic peptides (99, 101, 102); (2) modulating the intestinal permeability (45, 102–104); (3) driving the proper formation and differentiation of mucosal epithelium (99, 105); (4) regulating the expression of pro-inflammatory or anti-inflammatory cytokines (99, 106).

Several groups have investigated the composition of fecal, duodenal, and salivary microbiota in patients with CD. The main findings from these studies highlighted a reduction of *Lactobacilli* in both fecal and duodenal samples from active CD patients as compared to CD subjects following a GFD and healthy controls (107) as well as an increased abundance of *Bacteroides spp, E. Coli*, Proteobacteria, *Staphylococcus* and a decrease in *Bifidobacterium spp* (108).

While dysbiosis has been correlated with the active state of CD, our group and others have also shown that, in genetically predisposed infants, altered microbiome composition can precede the onset of the disease, therefore suggesting a possible causative role of dysbiosis in CD (109, 110).

Given the plasticity of the microbiota and its proven correlation with CD, the use of probiotics has been proposed to prevent and/or treat CD. Probiotics are described by the world health organization as “live microorganisms which, when administered in adequate amounts, confer a health benefit on the host.” Their protective role has been investigated by *in vivo* and *in vitro* studies, with the specific aim of finding a non-dietary therapy for a subset of CD patients that, despite being on GFD, have persistent symptoms.

An *in vitro* study performed by Lindforf et al. has shown that *Lactobacillus fermentum* and *Bifidobacterium lactis* reduce the toxic effects of gluten-derived peptides in intestinal CaCo2 cells by inhibiting the gliadin-induced intestinal permeability (111).

*In vivo* mice studies have shown promising results about the possible therapeutic effect of probiotic bacteria, such as *Lactobacillus casei*, on CD. This bacterium administered to immunized DQ8 mice was able to modulate both innate and adaptive immunity and to reduce gliadin-induced inflammation (112–114).

The use of probiotics has been suggested also for other gluten related disorders such as NCWS. Papista et al. have demonstrated that *Saccharomyces boulardii KK1* strain hydrolyzes gliadin toxic peptides, improves enteropathy and decreases histological damage as well as pro-inflammatory cytokine production in a model gluten-sensitive (114).

The study of Laparra et al. investigated the effect of *Bifidobacterium longum* CECT7347 in an animal model of gliadin-induced enteropathy and showed the ability of this strain to reduce the production of pro-inflammatory cytokines and their mediated immune response (115).

Work by D'Arienzo et al. has shown that the effect of probiotics is specific (116). In his work, the author demonstrated that *Lactobacillus* and *Bifidobacterium lactis* in transgenic mice expressing human DQ8 increased production of antigen-specific tumor necrosis factor (TNFα), therefore showing that certain strains of probiotics may have pro-inflammatory effects rather than beneficial ones.

De Angelis and his collaborators have analyzed the properties of probiotic preparation VSL#3, a mix of 8 strains including *Bifidobacterium breve, B. longum, B.infantis, Lactobacillus plantarum, Lactobacillus aciddophilus, Lactobacillus casei, Lactobacillus delbrueckii*, and *Streptococcus*. VSL#3 was able to decrease, *in vitro*, the toxic effects of wheat flour during the process of prolonged fermentation. This study pointed out that this probiotics mix was highly effective in hydrolyzing gliadin polypeptides compared to other commercial products (117) Moreover, it was also reported that the capability of VSL#3 to degrade gliadin was disabled if the probiotic strains were tested individually. These findings suggested that a single probiotic strain may not be sufficient to degrade gliadin peptides therefore highlighting the importance of using a combination of strains in order to obtain an effect against CD (117).

To this day, studies concerning the usage of probiotics in human are still limited. Smecuol et al. investigated the effects of *Bifidobacterium infantis* in a randomized study where 22 patients with active CD received the probiotic or placebo while on a gluten challenge diet for 21 days with a consumption of 12 g of gluten (118). The study showed that the *Bifidobacterium* strain was able to improve GI symptoms while no effect on cytokines production or celiac serology was reported.

In another randomized control trial, children newly diagnosed with CD were given *Bifidobacterium longum CECT 7347* for 3 months, while following a GFD. This probiotic showed improvements compared to placebo treatment, as well as lower peripheral CD4^+^ T lymphocytes concentration and slightly reduced TNFα. In addition, the treatment with NLS-SS significantly decreased *Bacteroides fragilis* and *Enterobacteriaceae* species. Improvements of gastro-intestinal symptoms were not reported (119).

A trial including 49 CD children evaluated the efficacy of a 3 months administration of 2 *Bifobacterium breve* in re-establish eubiosis in CD children on GFD. This study showed an increase of Actinobacteria as well as a restoration of the Firmicutes/Bacteroidetes ratio, (120). The same strains were investigated by Klemenak et al. the authors evaluated the effect of these probiotics on cytokines production and demonstrated that these specific bacteria lowered the levels of TNFα after 3 months of daily use, while no difference were found in IL10 production (121).

In 2018, Primec et al. performed a double-bind placebo-controlled study enrolling 40 CD and 16 healthy children. CD children were randomized to receive placebo or a mixture of two strains of *Bifidobacterium breve* for 3 months. They performed cytokines profiles analysis, CD serological markers (EMA, tTG) evaluation and clinical examination at three different points (at T(0): enrollment, at T(1): intervention with probiotic, at T(2): 3 months after intervention period. The probiotic mixture was able to modulate, in feces, the production of acetic acid and total short-chain fatty acids (SCFAs) (122).

Another randomized, double blind placebo controlled study evaluated the effect of *Bifidobacterium infantis* Natren life start strain (NLS-SS) on gut permeability, the occurrence of symptoms, and the presence of inflammatory cytokines in untreated CD patients. The study showed that the probiotic had no effect on modifying gut barrier function, but after 3 weeks it triggered an improvement in digestion process and reduction of constipation. Moreover, no differences in inflammatory markers were observed in either of the groups (118).

## Conclusions

Following a GFD represents the gold standard treatment for most patients affected by CD and NCWS, however a lifelong elimination of gluten from the diet can have a strong impact in patients' quality of life and it does not improve symptoms in a growing subpopulation of patients affected by RCD. Hence the importance of findings new non-dietary preventive therapies and treatments is becoming a subject of interest among the scientific community.

The pathogenesis of CD has been well-studied and numerous targets for adjuvant therapies have been already proposed. Although promising, the majority of therapies tested in clinical trials have shown important limitations. Probiotics, intestinal barrier modulators and endopeptidases appear to be successful in preventing damaging effects of small amounts of gluten, therefore addressing only the problem of cross contaminations, rather than representing a real alternative to GFD. Other therapies such as HLA-, TG2-inhibitors, and immunotherapies may have important side effects and more long-term studies are needed to better explore their usage and safety.

Given the heterogeneity of CD patients, a more stratified approach in which patients are divided in subgroups depending on clinical presentation, severity of symptoms, and response to GFD may be useful to work toward a more “personalized” therapy.

Overall, given the limitations of the proposed therapies and the proven efficiency of GFD in relieving symptoms from a high percentage of CD patients, a more realistic short-term goal could be to find adjuvant treatments targeting specific subsets of patients such as the ones with RCD or NRCD for which GFD alone has not been proven efficient.

The exact steps that lead to NCWS development are still unknown, therefore making the identification of therapeutic targets for this condition more difficult to achieve. The few clinical trials performed on NCWS patients are exploring the effect of endopeptidases and probiotics and aim at reducing the toxicity of the external trigger composition rather than specifically target the pathogenic cascade. More studies investigating the pathogenesis of NCWS are needed to find more effective treatments.

## Author Contributions

GS and PD'A wrote the manuscript. AF read and reviewed the manuscript. All authors contributed to the article and approved the submitted version.

## Conflict of Interest

AF is co-founder and stock holder of Alba Therapeutics. The remaining authors declare that the research was conducted in the absence of any commercial or financial relationships that could be construed as a potential conflict of interest.
